# Side effects of working from home: the dangers affecting the well-being of employees

**DOI:** 10.47626/1679-4435-2024-1178

**Published:** 2024-08-05

**Authors:** Josef Finsterer, Fulvio A Scorza

**Affiliations:** 1 Neurology and Neurophysiology Center, Vienna, Austria; 2 Disciplina de Neurociência. Universidade Federal de São Paulo/Escola Paulista de Medicina (UNIFESP/EPM). São Paulo, Brazil

We read with interest the article by Ogata et al. about a cross-sectional online survey of employees working from home, which was conducted by a Brazilian human resources website from June 1 to August 15, 2020.^1^ There was a total of 653 valid respondents, and a high prevalence of musculoskeletal symptoms, sleep disturbances, fatigue, headache, and migraine was reported among them.^1^ Low scores on the World Health Organization-5 Well-Being Index were significantly correlated with musculoskeletal symptoms, fatigue, headache, migraine, heartburn, indigestion, and leg pain.^1^ The study is compelling but has concerning limitations that should be discussed.

The main limitation of the study is the use of an online survey, which raises concerns about the accuracy of responses, whether the responses were given by the addressees themselves, and whether all respondents interpreted the questions in the same way. Another bias results from the fact that the survey was conducted by the operators of a website and not by the authors of the study.

Several factors that may impair the well-being and health of those working from home were not included in the study, such as the fact that working from home is usually associated with reduced social contact. Daily contact with colleagues or personal contact with customers, clients, or patients during work is seen as beneficial by many employees. Some employees even need contact with others at work for self-affirmation and self-empowerment.

Another unassessed factor is that working from home may be associated with increased tension between family members. Relationships often work because partners only see each other for a limited number of hours per day. Spending the entire day together while working from home can put a strain on the relationship.

Another factor not evaluated is the effect of electrosmog, to which these workers are likely exposed. There is a dearth of studies on the effect of electromagnetic fields on individual health, but most of them show that electromagnetic smog can impair well-being and health.^2^

Working from home may also expose employees to more manipulative information available on the Internet. Inevitably, workers are more widely exposed to advertising and other manipulative information at home than in a typical workplace, as there is no one at home to monitor their Internet use. Increased exposure to the Internet can not only reduce concentration, but also affect mood, motivation, and the way we think and feel.

As the evaluated symptoms could be a manifestation of depression, it would have been useful to use a depression scale to assess whether individuals working from home had any depressive symptoms due to changes in working conditions.

Overall, the study is interesting but has limitations that call its results and interpretation into question. Addressing these issues would strengthen the conclusions and could improve the quality of the study. There are several factors in addition to those evaluated that can strongly affect the well-being and health of those working from home. These factors need to be considered before drawing final conclusions on the effect of working from home on mental and physical health.
